# Orientation-dependent fiber-optic inclinometer based on core-offset michelson interferometer

**DOI:** 10.1038/s41598-022-12089-5

**Published:** 2022-05-12

**Authors:** Huajie Wang, Laifang Zheng, Junsheng Zhang, Jijun Liu

**Affiliations:** Department of Electronic Engineering, Taiyuan Institute of Technology, Taiyuan, 030000 China

**Keywords:** Engineering, Optics and photonics, Physics

## Abstract

An in-fiber Michelson interferometer (MI)-based inclinometer, which consists of misalignment-spliced fiber with end coating, is proposed and experimentally demonstrated. The incident light divided at the misalignment-spliced joint is reflected at the end coating, and then re-coupled into the fiber core. Due to the phase difference between the core mode and the $m{\text{th}}$ cladding mode, a typical MI is formed. The fiber near the misalignment-spliced joint is inserted in two capillary quartz tubes. The tilt of the capillary quartz tube leads to a significant deformation and curvature of the misalignment-spliced joint, which causes the wavelength and intensity of the MI spectrum to change. The experimental results indicate a good response within the angle range of 0°–50°. Both the wavelength modulation and intensity modulation are realized, with sensitivities of 0.55 nm/deg and 0.17 dB/deg, respectively. Moreover, the sensor shows a strong orientation dependence due to the asymmetric structure in the misalignment-spliced joint.

## Introduction

Tilt sensors (also known as inclinometers) are widely used in many applications, including structural health monitoring, landslide prediction, geotechnical/civil measurement and gesture sensing. In recent years, fiber-optic inclinometers are of great interest due to their unique advantages of compact size, remote monitoring, insensitivity to flammable and explosive gasses^1,2^, etc. Various schemes of fiber-optic inclinometers have been developed, which can be mainly classified into two types: the grating-based and the interferometer-based^3–6^. Take fiber Bragg grating (FBG) as an example, pre-processing such as polishing and tapering can couple light from the core to the cladding, making the FBG sensitive to angle change^7–9^. However, the pre-processing weakens the mechanical strength of the fiber, and the sensors usually suffered from instability. Moreover, both tilted fiber Bragg grating (TFBG) and long period fiber grating (LPFG) are typical structures to couple core mode to the cladding mode, which can be applied as inclinometers^10–12^. However, the sensing characteristics based on the transmission spectrum limit their application where a single-ended probe would be preferable (in vivo applications, for instance)^13^. As another type of fiber-optic device, in-fiber interferometers with separate interference arms are also sensitive to tilt^14^. For example, the fused taper is employed for the coupling of core-to-cladding modes, in which the interference spectrum is obtained^15^. As the tapered region is soft and easy to bend, many inclinometers based on fused taper have been proposed^16–18^. The drawback of this structure is that the fused taper is easy to be broken and the measurement range is small. In addition, different fibers with core-mode-field misalign are also spliced to form an interferometer for tilt measurement, such as thin-core fiber^19^, hollow-core photonic crystal fiber^20,21^, multicladding fiber^22^, etc. This type of sensor exhibits high sensitivity, however, these special fibers are expensive and the manufacturing process is complicated^23^.

In this paper, we propose and experimentally demonstrate a Michelson interferometer (MI)-based inclinometer using a simple configuration: a misalignment-spliced single mode fiber (SMF) with end coating. Different from the reported misalignment-spliced structures used for curvature^24^, bending^25,26^, and strain measurement, the proposed sensing probe is inserted in two capillary quartz tubes near the misalignment-spliced joint. The tilt of the quartz tube changes the coupling ratio between the core mode and the cladding modes, resulting in the shift of the reflection spectrum. In this way, Highly sensitive tilt measurement is achieved. The inclinometer shows a high sensitivity of 0.55 nm/deg, which makes it a good candidate for tilt measurement.

## Sensing system and thoretical analysis

**Figure 1:**
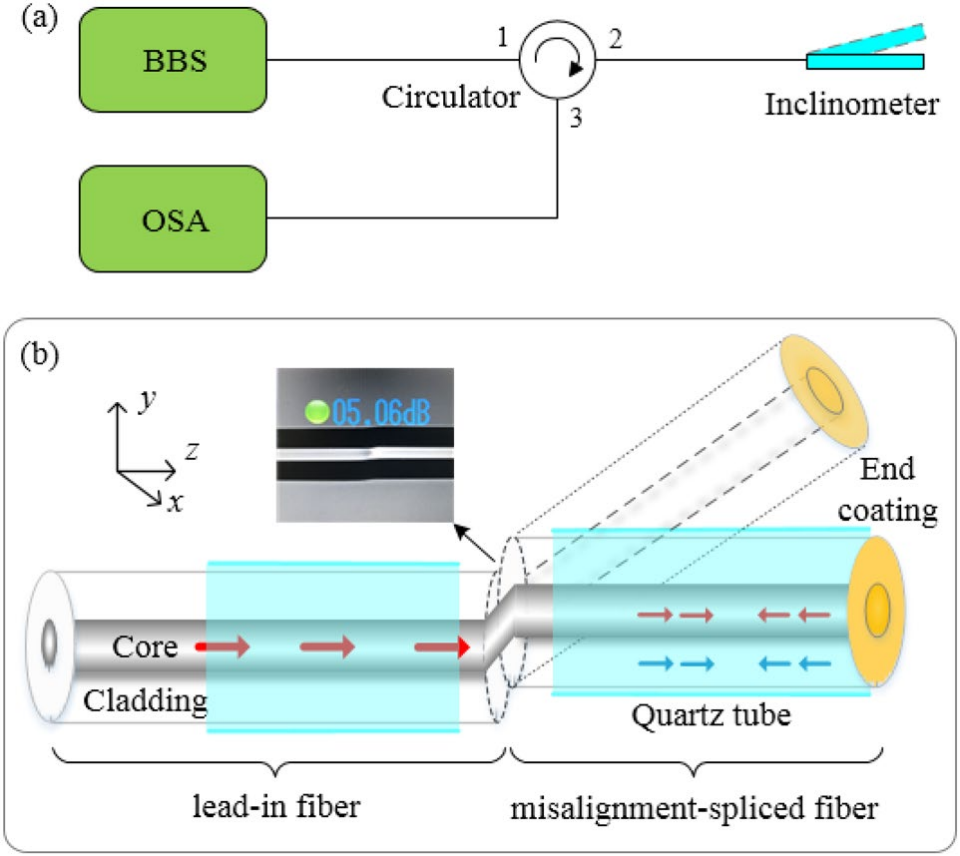
(**a**) Schematic diagram of the experimental setup. (**b**) Schematic diagram of the inclinometer.

The schematic diagram of the experimental setup is shown in Fig. 1a. A broad band source (BBS), an optical spectrum analyzer (OSA), a polarization controller (PC), and an optical circulator are employed to monitor the interference spectrum. The polarization state of the incident light is controlled by the three-ring PC. Figure 1b shows the schematic diagram of the MI-based inclinometer. The fiber axis is defined as the z-axis. Two sections of fiber are misaligned spliced, where the y-axis is offset and the x-axis is aligned. The left side of the misalignment-spliced joint is defined as “lead-in fiber”, and the right side is defined as “misalignment-spliced fiber”, as indicated in Fig. 1b. A copper film is plated on the end of the misalignment-spliced fiber. The lead-in fiber and the misalignment-spliced fiber are inserted in two capillary quartz tubes, respectively. The distance between these two capillary tubes is a few millimeters. The left capillary tube is fixed and the right capillary tube could be rotated freely so that the fiber angle can be adjusted. The image of the misalignment-spliced joint is shown in Fig. 1b.

As shown in Fig. 1b, the incident light from the lead-in fiber is divided into two parts at the misalignment-spliced joint. A part of the light is coupled into the core of the misalignment-spliced fiber as a core mode, and another part is coupled into the cladding of the misalignment-spliced fiber as cladding modes. These two parts of light are reflected at the copper film, and then re-coupled into the core and cladding of the lead-in fiber at the misalignment-spliced joint. The re-coupled core mode and the cladding modes in the core of the lead-in fiber are used for angle sensing.

Due to the phase difference of the re-coupled core mode and the cladding modes, a typical MI is formed in the core of the lead-in fiber. The interference fringe can be observed in the reflection spectrum, which can be expressed as:

1 $$ I = (I_{{{\text{co}}}} + \sum\limits_{m} {I_{cl}^{m} } + \sum\limits_{m} 2 \sqrt {I_{{{\text{co}}}} I_{cl}^{m} } \cos \varphi^{m} ) \cdot R, $$

where $I_{co}$ and $I_{cl}^{m}$ are the light intensity of the core mode and the $m{\text{th}}$ cladding mode. $R$ is the reflectivity of the copper film, and its value is usually greater than 0.8. $\varphi^{m}$ is the phase difference between the core mode and the $m{\text{th}}$ cladding mode, which can be written as:

2 $$ \Delta \varphi = {{2\pi (n_{eff}^{co} L_{co} - n_{eff}^{m,cl} L_{cl}^{m} )} \mathord{\left/ {\vphantom {{2\pi (n_{eff}^{co} L_{co} - n_{eff}^{m,cl} L_{cl}^{m} )} \lambda }} \right. \kern-\nulldelimiterspace} \lambda }, $$

where $L_{co}$ and $L_{cl}^{m}$ are the propagating length of the core mode and the $m{\text{th}}$ cladding mode. $\lambda$ is the incident light wavelength of the MI. $n_{eff}^{co}$ and $n_{eff}^{m,cl}$ are the effective refractive indexes of the core mode and the $m{\text{th}}$ cladding mode, respectively. When the phase difference satisfies the condition:

3 $$ \varphi^{m} = (2m + 1)\pi ,{\kern 1pt} {\kern 1pt} {\kern 1pt} {\kern 1pt} {\kern 1pt} {\kern 1pt} {\kern 1pt} {\kern 1pt} {\kern 1pt} {\kern 1pt} m = 0,1,2,3 \ldots , $$

a transmission dip appears at:

4 $$ \lambda_{m} = {{2(n_{eff}^{co} L_{co} - n_{eff}^{m,cl} L_{cl}^{m} )} \mathord{\left/ {\vphantom {{2(n_{eff}^{co} L_{co} - n_{eff}^{m,cl} L_{cl}^{m} )} {(2m + 1)}}} \right. \kern-\nulldelimiterspace} {(2m + 1)}}, $$

when the capillary quartz tube is bent and tilted, the incident angle of light at the misalignment-spliced joint is changed, so the phase difference between the core mode and the $m{\text{th}}$ cladding mode is changed accordingly. In this way, a wavelength shift of the interference spectrum has occurred. In addition, as the curvature of the misalignment-spliced fiber increases, more light is coupled from the core to the cladding, so the intensity of the interference spectrum is reduced accordingly.

## Sensor fabrication

Two sections of SMF (Corning, SMF-28e) are inserted in two capillary quartz tubes, and then, misaligned spliced using a commercial fusion splicer (FURUKAWA, S178) in a customized mode. The diameters of the fiber core and cladding are 9.2 μm and 125 μm, respectively. A copper film is plated on the cleaved end of the misalignment-spliced fiber using magnetron sputtering (ULVAC, ACS-4000-C4). The film thickness is 60 nm. The quartz tube is fixed to the fiber by epoxy resin adhesive (Henkel, E-120HP). The distance between these two capillary tubes is ~ 3.5 mm.

According to the Michelson interference theory, the fringe contrast depends on the coupling ratio and transmission loss of light. The degree of the core offsets affects the coupling ratio, and the length of the misalignment-spliced fiber affects the transmission loss of the core mode and the cladding modes. Therefore, it is important to improve the length of the misalignment-spliced fiber and the core offset of the splice joint, so as to enhance the fringe contrast.

**Figure 2:**
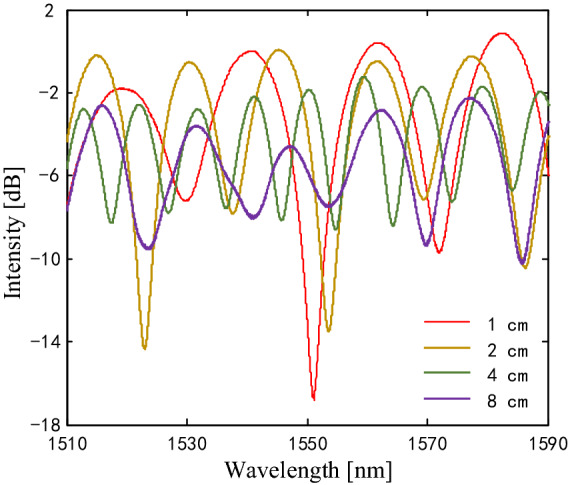
Interference spectra of the four MI samples with core offset of 6 μm and different lengths of 1, 2, 4, and 8 cm.

Four MI samples with a core offset of 6 μm and different lengths of 1, 2, 4, and 8 cm are fabricated. Each MI sample exhibits distinct fringe contrast and free spectrum range in the interference spectrum, as shown in Fig. 2. It can be found that the MI sample with a shorter fiber length has smaller fringe contrast. The MI sample with a fiber length of 1 cm has the largest fringe contrast. However, the short fiber is difficult to operate on the fusion splicer, so the misalignment-spliced fiber with a length of 2 cm is selected in the following experiment.

**Figure 3:**
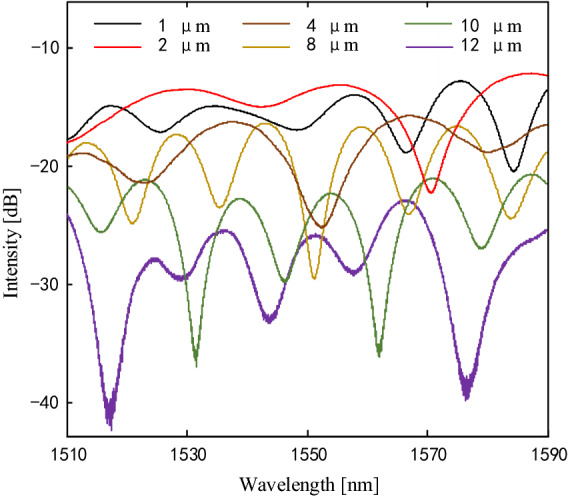
Interference spectra of the MI samples with length of 2 cm and core offsets of 1, 2, 4, 8, 10 and 12 μm.

Furthermore, the core offset of the misalignment-spliced joint is improved by moving the x- and y-directional motors in the fusion splicer. Six MI samples with a fiber length of 2 cm and different core offsets of 1, 2, 4, 8, 10, and 12 μm are fabricated. The interference spectra are shown in Fig. 3. The MI sample with an offset of 12 μm gets the maximum contrast ratio but endures large insertion loss. So there is a trade-off between the core offset and insertion loss. In the following experiment, the core offset of the misalignment-spliced joint is selected to be 8 μm.

## Experiment and discussion

**Figure 4:**
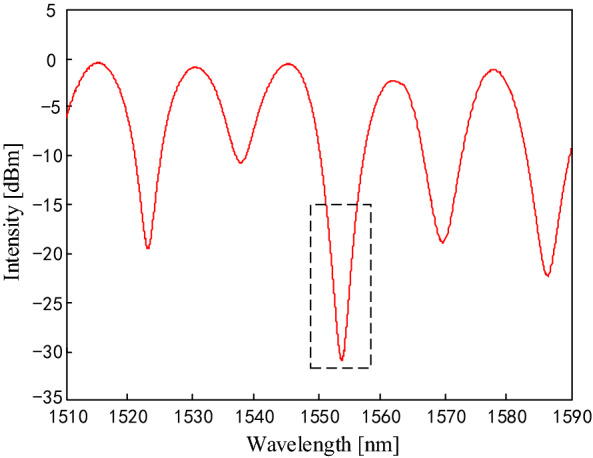
Interference spectrum of the sensor with misalignment-spliced fiber length of 2 cm and core offset of 8 μm.

With the experimental setup depicted in Fig. 1a, the tilt performance of the inclinometer is characterized. The optimized parameters, i.e. misalignment-spliced fiber length of 2 cm and core offset of 8 μm are selected. As shown in Fig. 4, the interference spectrum exhibits a fringe contrast of about 12 dB. The insertion loss is about − 31 dB, which is approximate to other fiber devices based on the Sagnac interferometer (about − 42 dB) and Mach–Zehnder interferometer (about − 32 dB)^27^. The wavelength and fringe contrast of the interference valley near 1553.2 nm is used as indicator for the tilt measurement, as shown in the dashed box in Fig. 4.

**Figure 5:**
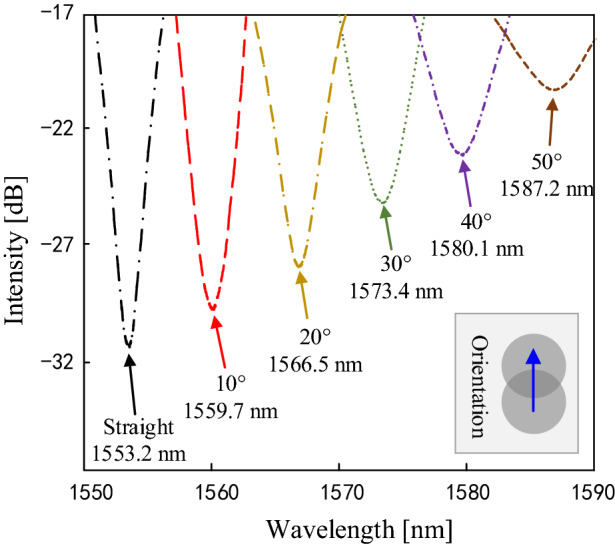
Some interference valleys of the inclinometer with the angle from 0° and 50°.

The experiment is carried out at constant room temperature (22.5 °C). The tilt angle is varied from 0° to 50°, with a step of 5°. An electronic angle meter (RION, DMI410) with an accuracy of 0.05° is used to provide a standard angle. The interference valleys with increasing angle are shown in Fig. 5, which show the strong angle feature. The angle orientation is along the y-axis, as shown in the inset.

**Figure 6:**
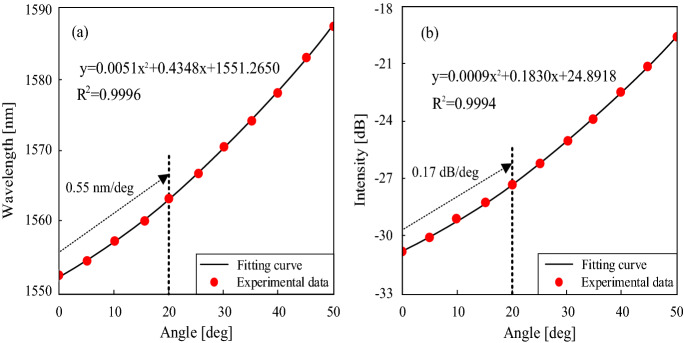
The wavelength (**a**) and intensity (**b**) response with respect to different tilt angles.

The wavelength and intensity of the interference valleys are plotted as the function of angle, as shown in Fig. 6a,b, respectively. When the angle increases from 0° to 50°, the interference valley shifts toward the longer wavelength, and the intensity increases. It is obvious that the response of the sensor to the angle is non-linear. It is because that more light is coupled from the core to the cladding as the angle becomes larger, and the cladding modes are more sensitive to surrounding bending. Moreover, the misalignment-spliced joint endures an extremely large strain in the case of a large angle, which increases the phase difference between the core mode and the high-order cladding mode. The response curves of the wavelength and intensity are obtained by quadratic fitting, the fitting equation is given in the figure. In the range of the tilt angle from 0° to 20°, The response of the sensor shows good linearity. The wavelength sensitivity of 0.55 nm/deg and intensity sensitivity of 0.17 dB/deg are obtained.

Although there is a three-ring PC in Fig. 1a, it should be noted that the experimental results are not affected by the polarization state in the angle range of 0°–50°. Only when the tilt angle is further increased (usually greater than 70°), the experimental results are affected by the polarization state. A new dip will emerge in the shorter wavelength region without a polarization controller. This phenomenon is consistent with Reference 28 and is caused by changes in the polarization state.

**Figure 7:**
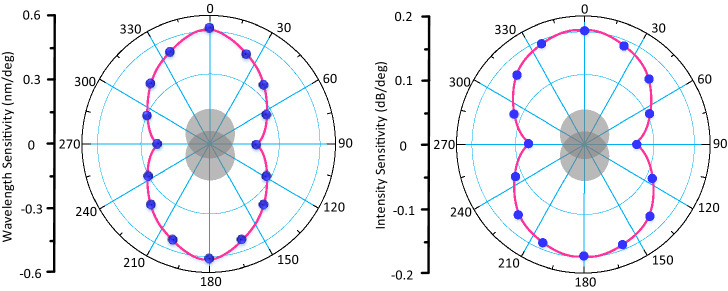
Angular dependence of the inclinometer with respect different orientations.

To study the orientation dependence of the inclinometer, the angle response over the orientations ranging from 0° to 360° are demonstrated. There are four layers of sheath on the lead-in fiber 150 mm away from the misalignment-spliced joint. The fiber and these sheaths are firmly bonded with epoxy resin adhesive. The outer diameter of the outermost sheath is 6 mm. Graduations at 20° intervals are marked around the outermost sheath. In this way, the orientation of the lead-in fiber can be determined according to the graduation. When the fiber is placed on the splicer, rotate the sheath to ensure that the 0° scale is on the top, so that the orientation of the misalignment-spliced joint can be determined. Figure 7 plots the wavelength sensitivity and intensity sensitivity at different orientations, which clearly shows the strong orientation dependence of the inclinometer. The angle response in all orientations is non-linear, similar to Fig. 6. The data in the linear range of 0°–20° is used to indicate the sensitivity in the corresponding orientation in Fig. 7.

As can be seen from Fig. 7, the maximum sensitivity is at the y-axis, and the minimum sensitivity is at the x-axis. This is easy to understand since the y-axis orientation has the largest misalignment at the misalignment-spliced joint, whereas the x-axis orientation has little misalignment. There are still some errors in Fig. 7, which may result from the following reasons: the two capillary tubes are incompletely set on the same horizon plane due to the fiber off-set, which may add a walk-off angle to the tilt; the mechanical turning of the sensor may bring in errors. For the in-field applications, the ambient temperature fluctuation always leads to sensor performance deterioration and large measurement error.

**Figure 8:**
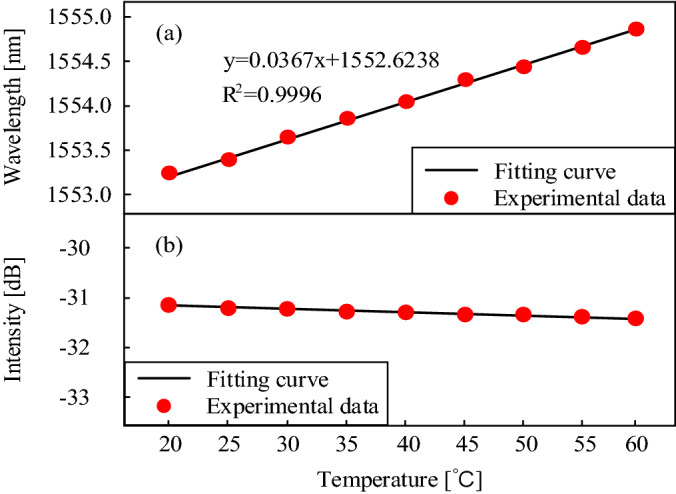
Temperature response of the inclinometer from 20 to 60 °C.

The temperature response of the proposed inclinometer is characterized. The inclinometer is placed in a tube furnace (SIGMA, OTF60). The temperature in the furnace rises gradually from 20 to 60 °C with a step of 5 °C and is maintained at each temperature for ~ 30 min. A thermometer (FLUKE, 1551A) with an accuracy of ± 0.05 °C is used to monitor the temperature in the tube furnace in real-time. The spectrum is collected when the temperature is stable. As shown in Fig. 8a, the wavelength of the interference valley shifts linearly toward the longer wavelength with a sensitivity of 36.7 pm/°C. The spectral shift is caused by the difference of effective refractive index between the core mode and the cladding modes, which is induced by the temperature rise^29^. Since the wavelength increases linearly, the temperature influence can be eliminated effectively by introducing a commercial FBG. Figure 8b shows the intensity of the interference valley at different temperatures. The maximum intensity variation over the whole heating process is less than 0.42 dB. It means that the temperature-angle cross-sensitivity of intensity demodulation is only ~ 0.062 deg/°C.

## Conclusion

The in-fiber interferometer based on misalignment-splicing is a classic structure for curvature, bending, and strain measurement. In our research, a compact and reflective in-fiber MI-based inclinometer is demonstrated. The proposed device is fabricated by means of misalignment-splicing and end coating. The fiber is inserted in two capillary quartz tubes near the misalignment-spliced joint, which makes it highly sensitive to tilt angle. The inclinometer presents a strong orientation dependence due to the asymmetric structure. The temperature response of the inclinometer is also characterized, and temperature compensation can be used to eliminate the temperature-induced error. The inclinometer has the advantages of compact size, high sensitivity and can provide remote sensing as a reflection probe, making it a good candidate for tilt measurement in many applications.

## References

[1] Li F, Zhang WT, Li F, Du YL (2012). Fiber optic inclinometer for land-slide monitoring. Appl. Mech. Mater..

[2] Zhuang Y (2020). A high-resolution 2-D fiber optic inclinometer for structural health monitoring applications. IEEE T. Instrum. Meas..

[3] Lee YG, Jang HK, Kim DH, Kim CG (2012). Development of a mirror Mounted fiber optic inclinometer. Sensor. Act. A. Phys..

[4] Li J, Qiao X, Rong Q, Sun A (2016). A compact fiber inclinometer using a thin-core fiber with incorporated an air-gap microcavity. F. I. G..

[5] Deng M, Tang CP, Zhu T, Rao YJ (2011). Highly sensitive bend sensor based on Mach-Zehnder interferometer using photonic crystal fiber. Opt..

[6] Mao L, Lu P, Lao Z, Liu D, Zhang J (2014). Highly sensitive curvature sensor based on single-mode fiber using core-offset splicing. Opt. Laser technol..

[7] Guan BO, Tam HY, Liu SY (2004). Temperature-independent fiber Bragg grating Tilt sensor. IEEE photon. Technol. Lett..

[8] Rauf A, Zhao J, Jiang B, Jiang Y, Jiang W (2013). Bend measurement using an etched fiber incorporating a fiber bragg grating. Opt. Lett..

[9] Rong Q (2012). Simultaneous measure-ment for displacement and temperature using fiber bragg grating cladding mode based on core diameter mismatch. J. Lightwave. Technol..

[10] Albert J, Shao LY, Caucheteur C (2012). Tilted fiber bragg grating sensors. Laser. Photonics. Rev..

[11] Guo C, Chen D, Shen C, Lu Y, Liu H (2015). Optical inclinometer based on a tilted fiber Bragg grating with fused taper. Opt. Fiber. Technol..

[12] Frazão O (2006). Optical inclinometer based on a single long-period fiber grating combined with a fused taper. Opt. Lett..

[13] Shao LY, Albert J (2010). Compact fiber-optic vector inclinometer. Opt. Lett..

[14] Li J, Qiao X, Rong Q, Sun A (2016). A compact fiber inclinometer using a thin-core fiber with incorporated an air-gap microcavity fiber interferometer. Sensors..

[15] Osuch T, Markowski K, Manujło A, Jędrzejewski K (2016). Coupling independent fiber optic tilt and temperature sensor based on chirped tapered fiber Bragg grating in double-pass configuration. Sen. Actuators A. Phy..

[16] Lee C (2020). Tapered polymer fiber inclinometers. IEEE Photon. J..

[17] Feng ZY (2016). A fiber inclinometer using a fiber microtaper with an air-gap microcavity fiber interferometer. Opt. Commun..

[18] Gong H, Qian Z, Yang X, Zhao CL, Dong X (2015). Optical fiber inclinometer based on a fiber taper cascading a peanut-shape structure. IEEE Sen. J..

[19] Li, J., Qiao, X., Rong, Q. & Sun, A. A Compact fiber inclinometer using a thin-core fiber with incorporated an air-gap microcavity fiber interferometer. *Sensors*. **16** (1), (2016). 10.3390/s16010092 PMC4732125 26771614

[20] Liu S (2013). Direction-independent fiber inclinometer based on simplified hollow core photonic crystal fiber. Opt. Lett..

[21] Gong H, Song H, Zhang S, Jin Y, Dong X (2014). Curvature sensor based on hollow-core photonic crystal fiber sagnac interferometer. IEEE Sens. J..

[22] Qi Y (2014). Highly sensitive curvature Sensor based on a multicladding fiber sandwiched dual no-core fibers structure. Appl. Opt..

[23] Smart Sensor Technology and Measurement Systems (2002). Inaudi, D. & Glisic, B. Development of a fiber optic interferometric inclinometer. Smart Structures and Materials. 2002. Int. Soc. Opt. Photon..

[24] Mao L, Lu P, Lao Z, Liu D, Zhang J (2014). Highly sensitive curvature sensor based on single mode fiber using core-offset splicing. Opt. Laser. Technol..

[25] Wang LY (2016). Bending vector sensor based on Machzehnder interferometer using S type fibre taper and lateral-offset. J. Mod. Optic..

[26] Zhang S, Zhang W, Gao S, Geng P, Xue X (2012). Fiber-optic bending vector sensor based on Mach-Zehnder interferometer exploiting lateral-offset and up-taper. Opt. Lett..

[27] Song B (2013). Orientation dependant inclinometer based on intermodal coupling of two-LP-modes in a polarization-maintaining photonic crystal fiber. Opt. Express..

[28] Song B (2013). Multi-mode interferometer-based twist sensor with low temperature sensitivity employing square coreless fibers. Opt. Express..

[29] Lu P, Men L, Sooley K, Chen Q (2009). Tapered fiber Mach-Zehnder interferometerfor simultaneous measurement of refractive index and temperature. Appl. Phys. Lett..

